# Zidovudine plus lamivudine in Human T-Lymphotropic Virus type-I-associated myelopathy: a randomised trial

**DOI:** 10.1186/1742-4690-3-63

**Published:** 2006-09-19

**Authors:** Graham P Taylor, Peter Goon, Yoshitaka Furukawa, Hannah Green, Anna Barfield, Angelina Mosley, Hirohisa Nose, Abdel Babiker, Peter Rudge, Koichiro Usuku, Mitsuhiro Osame, Charles RM Bangham, Jonathan N Weber

**Affiliations:** 1 Department of GU Medicine and Communicable Diseases, Faculty of Medicine, Imperial College, London, UK; 2 Department of Immunology, Faculty of Medicine, Imperial College, London, UK; 3 3^rd ^Department of Internal Medicine, University of Kagoshima, Kagoshima, Japan; 4 Clinical Trials Unit, Medical Research Council, London, UK; 5 The National Hospital for Neurology and Neurosurgery, London, UK

## Abstract

**Background:**

No therapies have been proven to persistently improve the outcome of HTLV-I-associated myelopathy. Clinical benefit has been reported with zidovudine and with lamivudine in observational studies. We therefore conducted a randomised, double blind, placebo controlled study of six months combination therapy with these nucleoside analogues in sixteen patients.

**Results:**

Primary outcomes were change in HTLV-I proviral load in PBMCs and clinical measures. Secondary endpoints were changes in T-cell subsets and markers of activation and proliferation.

Six patients discontinued zidovudine. No significant changes in pain, bladder function, disability score, gait, proviral load or markers of T-cell activation or proliferation were seen between the two arms. Active therapy was associated with an unexplained decrease in CD8 and non-T lymphocyte counts.

**Conclusion:**

Failure to detect clinical improvement may have been due irreversible nerve damage in these patients with a long clinical history and future studies should target patients presenting earlier. The lack of virological effect but may reflect a lack of activity of these nucleoside analogues against HTLV-I RT *in vivo*, inadequate intracellular concentrations of the active moiety or the contribution of new cell infection to maintaining proviral load at this stage of infection may be relatively small masking the effects of RT inhibition.

## Background

The first identified human retrovirus, Human T-cell Lymphotropic Virus Type I (HTLV-I) [1], was initially associated with Adult T-cell Leukaemia/Lymphoma[2] before becoming causally associated with a chronic inflammatory myelopathy (HTLV-I associated myelopathy, HAM)[3,4] in a minority of carriers. In Japan it has been estimated that the lifetime risk of HAM among the one million infected persons is 0.25%[5] whilst in England, the estimated 22,000 carriers, of mainly African descent, have a 3% lifetime risk[6].

The onset of HAM is usually sub-acute and the initial presenting symptoms may relate to the urinary bladder, bowels or sexual function as well as gait disturbance or back pain. Diagnosis is often delayed until the more characteristic constellation of combined symptoms appears some months or years later. The most rapid progression is usually seen within the first two years with the neurological deficit varying from mild gait impairment to paraplegia. Thereafter some patients remain relatively stable whilst slow deterioration compounded by physiological aging is observed in others. In our experience approximately half of all patients become wheel-chair dependant and although many patients with HAM have a normal life span, premature deaths directly related to HAM occur.

The pathogenesis of HAM is not fully understood but several observations point to potential therapeutic approaches. An association between proviral load and disease has been repeatedly found with proviral load, as measured by HTLV-I DNA copies in peripheral blood mononuclear cells (PBMCs), about ten fold higher in patients with HAM than in asymptomatic carriers[7,8] and the risk of disease increases exponentially if the proviral load is greater than one HTLV-I DNA copy per 100 PBMCs[9]. Thus measures to reduce viral burden could potentially reduce the risk, or modify the course of the disease. Neuropathological examination reveals a perivascular lymphocytic infiltration in the spinal cord that is composed predominantly of CD4 positive T-lymphocytes (CD4 cells) in early disease and CD8 positive T- lymphocytes (CD8 cells) in later disease followed by a less cellular, atrophic phase[10,11]. The same cell types can be found in the cerebrospinal fluid of patients with HAM[12]. Like other cytotoxic T-lymphocytes (CTL) HTLV-I specific CTL have been shown to release potentially neurotoxic cytokines such as interferon-γ (IFN-γ) and tumour necrosis factor-α (TNF-α)[13]. In the periphery HTLV-I-specific CD4 T cells differ much more in frequency between patients with HAM and asymptomatic carriers than do anti-HTLV CTL and these also secrete IFN-γ and TNF-α [14]. Thus therapies, which might modulate the immune response such as corticosteroids and interferons, have also been considered.

The literature on the specific management of HAM consists mostly of small, uncontrolled studies and cohort data. Conflicting results have been obtained in studies of corticosteroids therapy[15]. Interferon-α has been shown to be of short-term benefit in some patients in a randomised study comparing three different dose regimens with evidence of a dose dependant response[16]. Some nucleoside analogues have been shown to inhibit HTLV-I reverse transcriptase. The thymidine analogue, zidovudine, which inhibits HTLV-I RT in vitro[17,18] and in a rabbit infection model[19], was reported to show no clinical benefit in a study of six patients with chronic disease[20] but associated with improved mobility is some patients in a second study[21]. The cytosine analogue, lamivudine, was reported to reduce HTLV-I proviral load in five patients with clinical improvement in one patient with early disease[22]. All three studies were small, open and uncontrolled. In the management of both HIV and hepatitis B virus treatment with nucleoside analogues fails because of the emergence of viral strains with reduced sensitivity to these compounds especially when the drugs are used singly.

We report here the results of a randomised, double blind, placebo controlled study of the combination of zidovudine and lamivudine to determine their safety and efficacy with medium term (six to twelve months) usage.

## Results

Sixteen patients were recruited to the study, twelve in London and four in Kagoshima. Eight were randomly assigned to each study arm. Baseline demographics, clinical and laboratory characteristics of the participants are shown in Table 1. Treatment was initiated on the day of randomisation in seven participants, within two weeks for seven participants and after two weeks for two participants. All participants were followed up to week 48.

**Table 1:** Baseline characteristics

	Placebo (n = 8)	Active (n = 8)	Total (n = 16)
**Demographics**			
Country [n(%)]			
England	6 (75)	6 (75)	12 (75)
Japan	2 (25)	2 (25)	4 (25)
Gender [n(%)]			
Male	4 (50)	1 (13)	5 (31)
Ethnic origin [n(%)]			
Afro-Caribbean	5 (63)	5 (63)	10 (63)
Indian	1 (13)	0 (0)	1 (6)
Japanese	2 (25)	2 (25)	4 (25)
Persian	0 (0)	1 (13)	1 (6)
Age at randomisation			
mean years (SD)	61.0 (10.8)	53.9 (15.5)	57.4 (13.4)
Likely mode of infection^a ^[n(%)]			
Mother to child	3 (30)	5 (33)	8 (32)
Blood transfusion	1 (10)	2 (13)	3 (12)
Sexual intercourse	2 (20)	6 (40)	8 (32)
Unknown	4 (40)	2 (13)	6 (24)

**Clinical Characteristics**			
Pain score: scale 0–10			
median (range)	2.2 (0–5)	2.6 (0–8)	2.6 (0–8)
Osame's score [n(%)]			
0 – 4 (Unaided walk)	3 (38)	3 (38)	6 (38)
5 – 8 (Needs aid to walk)	4 (50)	3 (38)	7 (44)
9 – 13 (Unable to walk)	1 (13)	2 (25)	3 (19)
Time to walk 13 m in seconds [mean (SD)]			
0 – 4 (Unaided walk)	19 (13)	13 (2)	16 (9)
5 – 8 (Needs aid to walk)	82^b ^(65)	112 (112)	95^b ^(81)
Bladder function^c ^[median (range)]			
Daytime frequency	5 (4–8)	4.5 (2–8)	5 (2–8)
Nocturia	4 (3.5–4)	2 (0.5–4)	4 (0.5–4)
Duration of symptoms in years			
median (range)	10.5 (5 – 19)	9 (1–18)	9 (1–19)

Laboratory Measurements*			
HTLV-I proviral load^d ^(log_10 _copies/10^5 ^PBMCs)			
mean (SD)	3.57 (0.44)	3.76 (0.39)	3.67 (0.41)
Total lymphocytes^e ^(10^6^/L)	1600 (1250–2600)	1800 (1250–3000)	1775 (1250–3000)
CD3 lymphocytes^e ^(10^6^/L)	1165(488–2018)	1240(430–2144)	1190(430–2144)
CD3^e ^%	71 (35–81)	69 (34–90)	71 (34–90)
CD4^e ^lymphocytes (10^6^/L)	802(340–1670)	801(334–1375)	801(334–1670)
CD4^e ^%	39 (25–67)	44 (27–59)	44 (25–67)
CD8^e ^lymphocytes (10^6^/L)	375 (149–941)	185 (96–840)	362 (96–941)
CD8^e ^%	27 (11–36)	10 (6–46)	21 (6–46)
CD25^d ^%	3 (2–26)	7 (2–33)	3 (2–33)
CD69^d ^%	8 (4–10)	7 (4–14)	7 (4–14)
CD71^d ^%	9 (3–12)	7 (3–18)	8 (3–18)
HLA-DR%	20 (13–40)	9 (5–16)	15 (5–40)

*median (range) unless otherwise specified

^a ^Total exceeds 16 because more than one possible mode of infection was documented in some patients

^b ^One patient could only walk half distance. For the analysis their timed walk was doubled

^c ^One patient in each group is excluded from the analysis because they had an indwelling urinary catheter.

A further 5 patients (3 in placebo group, 2 in active group) have missing nightly frequencies. Values given as average daily/nightly urinary frequency between screening and week 0

^d ^Baseline data only available on n = 6 in placebo group and n = 7 in active group

^e ^Baseline data only available on n = 7 in placebo group and n = 7 in active group

### Adherence

Mean red cell volume rose during the first 24 weeks of the study in the active arm. At week 24, 100% of patients in the active arm and 0% in the placebo arm had a change in MCV from baseline greater than 3 within-person SDs. During the second 24 weeks of the study a similar rise in MCV was seen in the placebo arm at week 48, 75% of patients in each arm had a change in MCV from baseline greater than 3 within-person SDs. There was good consistency between lamivudine and zidovudine returned tablet counts but the average number of tablets returned in the previous month rose from 9.5% at the end of the 3^rd ^month to 25.5% at the end of the 6^th ^month. There was little detectable difference in returned tablet counts between the two arms or between the first, randomised and second, open phases of the study.

### Safety

During the first 24 weeks of the study no participants in the placebo arm discontinued therapy. In the active arm one participant, whose disease had progressed from first symptom to bed-bound in the 9 months prior to study entry, was un-blinded at week 8 because of continuing deterioration. Interferon-α was added to the active compounds until week 20 when zidovudine was discontinued following the development of autoimmune haemolytic anaemia. During the second 24-week period of open therapy 5 participants previously on placebo discontinued zidovudine, one within 4 weeks, with gastrointestinal symptoms, the remainder after 16 – 20 weeks of therapy. Two had anaemia, one necessitating blood transfusion, one complained of drowsiness and paraesthesia and one of lethargy. One participant from the active therapy arm elected to discontinue therapy at week 40 when antibiotics were prescribed out of study. No side affects attributable to lamivudine were reported. There were no significant biochemical abnormalities.

### Clinical efficacy

**Figure 1:**
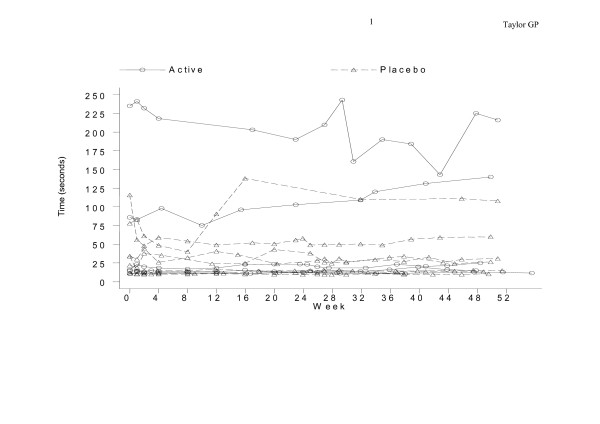
Changes in timed 13 meter walk observed during the study period.

No significant changes in pain score, urinary frequency or nocturia were seen between the active arm and the placebo arm during the study (Table 2) nor within the placebo arm when comparing the first 24 weeks on placebo with the second 24 weeks on active therapy. Similarly there was no consistent pattern of change in disability. Timed walks remained relatively constant throughout the study in most participants except for two in the placebo arm whose timed walks improved between the two baseline assessments and week 4 (Figure 1).

**Table 2:** Clinical outcome measures during randomised treatment phase presented as average [mean (SE)] changes from baseline during weeks 0 – 24 as measured by AUCMB

	Placebo (n = 8)	Active (n = 8)	p
Osame's score	+0.19 (0.19)	+0.18 (0.34)	0.99

Pain score	+0.43 (1.13)	+0.41 (0.66)	0.99

Bladder function			
Daytime frequency^a^	-0.11 (0.38)	-0.19 (0.62)	0.93
Nocturia^b^	-0.18 (0.55)	-0.81 (0.47)	0.41

^a ^One patient in each group is excluded from the analysis because they had an indwelling urinary catheter

^b ^One patient in each group is excluded from the analysis because they had an indwelling urinary catheter. The four Japanese patients had no night urinary frequency data (2 in each group) and baseline frequency was unknown for another patient in the placebo group. All are excluded from the analysis.

Laboratory markers of efficacy: The median (IQR) HTLV-I proviral load at baseline was 4.2 (2.6–7.5) copies per 100 PBMCs (mean (SD) 3.7 (0.4) log_10_/100,000 PBMCs). No significant change in proviral load was seen during the first 24 weeks of the study in the active arm compared with the placebo arm (mean difference (SE): 0.02 (0.17), p = 0.92) (Table 3) nor within the placebo arm comparing the first 24 weeks of placebo therapy with the second 24 weeks of active therapy (mean difference (SE): 0.12 (0.16), p = 0.50) (Table 4).

**Table 3:** Laboratory outcome measures during randomised treatment phase presented as average [mean (SE)] changes from baseline during weeks 0 – 24 as measured by AUCMB

	Placebo	Active	p
HTLV-I proviral load^a ^(log_10 _copies/10^5 ^PBMCs)	+0.03 (0.10)	+0.05 (0.13)	0.92
Total Lymphocytes^b ^(10^6^/L)	+148.2 (58.0)	-159.3 (146.7)	0.07
CD3 Lymphocytes^b ^(10^6^/L)	+98.4 (63.4)	-23.2 (151.3)	0.47
CD3^b ^%	+0.2 (2.8)	+4.3 (2.6)	0.14
CD4^b ^Lymphocytes (10^6^/L)	+32.6 (33.1)	+10.4 (98.7)	0.55
CD4^b ^%	-1.2 (0.9)	+4.7 (1.8)	0.01
CD8^b ^Lymphocytes (10^6^/L)	+65.8 (31.3)	-38.1 (60.7)	0.15
CD8^b ^%	+1.4 (1.2)	+1.4 (1.9)	0.99
CD25^a ^%	-0.8 (1.8)	+0.3 (0.8)	0.58
CD69^a ^%	+0.4 (1.3)	+1.2 (1.8)	0.73
CD71^a^%	-0.3 (1.0)	-0.2 (1.9)	0.97
HLA-DR%	-4.85 (4.0)	+3.59 (2.4)	0.42

^a ^Data only available on n = 6 in placebo group and n = 7 in active group due to missing baseline data

^b ^Data only available on n = 7 in placebo group and n = 7 in active group due to missing baseline data

**Table 4:** Laboratory outcome measures for the placebo group presented as average [mean (SE)] changes from baseline for the time on active therapy (weeks 24 – 48) compared with the time on placebo (weeks 0–24). Baseline values are the average of weeks – 2 and 0 for 0 – 24 week analysis and the average of weeks 20 and 24 for 24 – 48 week analysis

	Weeks 0–24	Weeks 24–48	P
HTLV-I proviral load^a ^(log_10 _copies/10^5 ^PBMCs)	+0.03 (0.10)	-0.09 (0.08)	0.50
Total Lymphocytes^b ^(10^6^/L)	+148.2 (58.0)	-71.1 (63.1)	0.02
CD3 Lymphocytes^b ^(10^6^/L)	+98.4 (63.4)	+7.0 (39.2)	0.28
CD3^b ^%	+0.2 (2.8)	+2.8 (2.0)	0.22
CD4^b ^Lymphocytes (10^6^/L)	+32.6 (33.1)	+30.4 (46.9)	0.97
CD4^b ^%	-1.2 (0.9)	+3.6 (1.9)	0.08
CD8^b ^Lymphocytes (10^6^/L)	+65.8 (31.3)	-23.5 (13.7)	0.04
CD8^b ^%	+1.4 (1.2)	-0.7 (1.1)	0.16
CD25^a ^%	-0.8 (1.8)	-0.2 (1.3)	0.82
CD69^a ^%	+0.4 (1.3)	-0.2 (3.0)	0.89
CD71^a^%	-0.3 (1.0)	+0.5 (1.3)	0.76
HLA-DR%	-4.85 (4.0)	+1.05 (2.3)	0.40

^a ^Data only available on n = 6 due to missing baseline data

^b ^Data only available on n = 7 due to missing baseline data

At baseline, the total lymphocyte, CD3, CD4 and CD8 cell counts were normal. During the first 24 weeks the mean total lymphocyte counts (AUCMB) increased by 148 × 10^6^/L in the placebo arm but decreased by 159 × 10^6^/L in the active arm, a mean difference of 307 × 10^6^/L (SE = 158, p = 0.07) (Table 3). This trend was confirmed when comparing the lymphocyte counts in participants on placebo compared with their counts when taking active therapy, with the initial increase of 148 × 10^6^/L followed by a decrease of 71 × 10^6^/L (mean difference (SE): 219 (73), p = 0.02) (Table 4). Lymphocyte subset analysis of the first, randomised therapy phase of the study, showed an increase in CD4 in both arms and an increase in CD8 cells in the placebo arm but a decrease in the active arm (Table 3). These changes were also seen when comparing the first 24 weeks of placebo treatment with the subsequent 24 weeks of active treatment in the placebo arm (Table 4). However the major impact on the absolute lymphocyte count appears mainly among non-T-lymphocytes with a mean rise of 50 × 10^6^/L non CD3 lymphocytes during the first 24 weeks in the placebo arm compared with a mean decrease of 127 × 10^6^/L from baseline in the active arm, a difference of 177 × 10^6^/L (SE = 31, p < 0.001). A similar trend was seen by comparing active with placebo therapy in the placebo arm, although not statistically significant.

At baseline CD69 was expressed on average on 8%, CD71 on 8% and CD25 on 9% of lymphocytes. Compared with placebo no significant changes were seen in these markers of activation (CD69) and proliferation (CD71) nor in the expression of the IL-2 receptor (CD25) (Tables 3 and 4) during treatment with zidovudine plus lamivudine.

## Discussion

Since monotherapy with nucleoside analogues had been reported to reduce HTLV-I viral load, the inhibition of HTLV-I reverse transcriptase by two nucleoside analogues, zidovudine and lamivudine was thought to be more likely to cause a sustained decrease in HTLV-I viral burden than either drug alone. A decrease in HTLV-I viral DNA and thus a presumed decrease in viral antigen burden has been associated with a reduction in HTLV-I specific CTL lymphocyte activity[22].

Since this activity is postulated to contribute to HAM pathogenesis anti-HTLV therapy might in turn delay progression or cause clinical improvement.

HAM is a rare disease in the UK with only 10 – 12 cases diagnosed annually[23]. A similar number of cases are seen in Kagoshima, Japan where the prevalence of infection is much higher. There is no licensed or clinically proven effective therapy for HAM in the UK. However short courses of interferon-α were made available by the Japanese government for their patients with HAM coincident with the start of the study and this affected recruitment in Japan.

Although the basic study design was double-blind and placebo-controlled a second open-therapy phase was incorporated, with all study participants offered open therapy for a further six months, to maximise the likelihood of detecting significant clinical and laboratory changes. The study was not un-blinded until the last participant had completed twelve months of therapy. In this way the safety of zidovudine plus lamivudine could be studied for a total of twelve months exposure whilst those originally randomised to the placebo arm acted as their own control for the second six months of active compound in a non-randomised comparison.

The primary outcome measures of the study were the clinical and virological effects of zidovudine in combination with lamivudine. The failure to detect any clinical improvement after up to 12 months of therapy may have been due to the long history of HAM among the participants who had an average duration of symptoms of 10.1 years. After this prolonged time the neurological damage may have been irreversible. However, the stability of the HTLV-I viral DNA load, which was expected to fall during the study, leaves the possibility that agents that effectively reduced viral burden, might result in clinical improvement even in longstanding disease.

The persistence of high viral load during therapy could be due to a lack of activity of these nucleoside analogues against HTLV-I RT, inadequate intracellular concentrations of the active triphosphate metabolite, or it may be that reverse transcription is not important in maintaining HTLV-I proviral load at this stage of infection. Thus, whilst a reduction in HTLV-I viral DNA during therapy with lamivudine has been documented in vivo, there are now conflicting reports of its activity against HTLV-I RT in vitro [24-26]. Conversely the in vitro inhibition of HTLV-I RT by zidovudine has been confirmed[27]. Second, the intracellular concentrations of the nucleoside analogues were not measured in this study but HTLV-I Tax, which is expressed within hours by the majority of HTLV-I infected cells in unstimulated culture conditions, is known to upregulate MDR-1[28]. Finally, the importance of HTLV-I RT in maintaining HTLV-I proviral load remains uncertain. Several paths of evidence point to continuing viral protein expression[29] and HTLV-I has been shown to spread directly from cell to cell, in ex vivo experiments, through the formation of a virus induced synapse[30]. Whether cell-to-cell infection within the host is predominantly through virion production, binding and fusion or directly through the viral synapse the reverse transcription of HTLV-I RNA is required to establish new infection. However HTLV-I infection is associated with proliferation of the infected cells, a mechanism that contributes to the total proviral load without the need of reverse transcription.

Changes in the lymphocyte markers of activation and proliferation might have been expected with a reduction in HTLV-I proviral load. In the absence of such an effect the observed, statistically significant, changes in total, CD4, CD8 and non-CD3 lymphocytes are of interest albeit unexplained.

## Conclusion

The data from this study do not provide evidence of improvement in the clinical state of patients with HAM in the medium term following treatment with the combination of zidovudine and lamivudine but the treatment was well tolerated with no unexpected side effects. New, controlled studies of both anti-viral and anti-inflammatory agents are urgently required for patients with this chronic, disabling disease. International co-operation will be necessary to accelerate progress.

## Methods

### Study design

Patients were screened two weeks prior to baseline and eligibility criteria checked. Recruitment was by the study physicians at each site. The eligibility form was submitted with a request for a trial number to the coordinating centre at the Medical Research Council. Trial numbers had been randomly allocated, stratified by site, to active or placebo and this code was held, sealed and secure, at the MRC. Trial therapy was stored in site pharmacies and labelled by trial name and trial number only. The trial coordination staff at the MRC issuing the trial number, the pharmacy, the clinician and the patient were all blinded to the allocation of study arm. Trial numbers were transmitted by facsimile to the study centres. Recruitment commenced 8 November 1999. Eligible patients were randomised to start 24 weeks treatment with zidovudine 300 mg plus lamivudine 150 mg twice daily (the active arm) or matching zidovudine and lamivudine placebo tablets (the placebo arm). This was followed by 24 weeks of open therapy with the active compounds for all study participants. Last follow up was completed 30 July 2002. The primary endpoints were virological – change in HTLV-I viral DNA from baseline to 24 weeks and clinical – changes in gait, disability, pain and bladder function. The secondary endpoints were immunological – changes in CD4, CD8, CD25, CD69 and CD71. The study, based on recruiting twelve patients in each arm and assuming a standard deviation of 0.78 log _10 _copies of HTLV-I DNA per 100 PBMCs, was powered to detect a difference in average change from baseline up to 24 weeks (as measured by area under the curve minus baseline – AUCMB) of 1 log_10 _copy with 90% power and 5% probability of type 1 error. Therapeutic safety was determined by monitoring standard haematological and biochemical parameters according to National Institute of Health, Division of AIDS criteria[31].

### Subjects

Patients were eligible for the study if they had clinical evidence of HAM according to WHO criteria [32], had confirmed HTLV-I infection, were aged 18 – 75 years, not pregnant and willing to use appropriate contraception if female and sexually active. Patients were excluded if they had HIV infection, had previous exposure to zidovudine or lamivudine, had significant haematology, liver or renal test abnormalities or were unable to give informed consent. Patients were not eligible to enter the study until at least four weeks after concluding other specific treatment for HAM e.g. corticosteroids. The study was conducted at two sites, The HTLV clinic at St. Mary's Hospital, London, UK and the 3^rd ^Department of Internal Medicine, University of Kagoshima, Kagoshima, Japan. At each site the local research ethics committee approved the study and written informed consent was obtained in the local language.

### Clinical evaluation

Participants underwent a full neurological examination at weeks 0, 24 and 48 and if new symptoms were reported. Participants were reviewed monthly with additional visits at weeks 1, 2, 25 and 26. At each visit a fixed distance walk was timed and the degree of walking aid documented for patients able to walk. Disability was graded 1 – 13 as described by Osame [15]. Pain was quantified using a visual analogue scale and urinary bladder diary cards recording daytime frequency and nocturia were collected. Participants were provided with 70 tablets of each compound or matching placebo every four weeks and any remaining tablets were returned and counted to provide a measure of adherence.

### Laboratory evaluation

Full blood counts, biochemical profile, quantitative HTLV-I viral DNA and lymphocyte phenotypic assays were performed at each study visit. The clinical investigators were blinded to the mean red cell volumes although these were available for the end of study analysis. Full blood counts were measured on a Coulter LH750 Analyzer (Beckman Coulter Inc, Fullerton, CA). The renal, liver and bone chemistry assays were performed on an AU2700 Olympus Analyser (Olympus Diagnostica Gmbh, Hamburg, Germany). HTLV-I viral DNA was quantified by real-time PCR as previously described. [33]. In addition to the standard T-lymphocyte markers CD3, CD4, CD8, markers of T-cell activation CD25, CD69 and proliferation CD71 were assayed described earlier [34].

### Statistical analysis

Data from clinical record files were entered into Oracle databases and checked manually and by computer consistency checks. Analysis text files were created from the database and imported into STATA for statistical analysis. Baseline values of laboratory tests were calculated as the mean of screening and week 0 results. Week 0 data were taken as baseline for all clinical measurements. Time was measured from the start of trial treatment and for all measurements during follow-up data from the closest date to the target assessment week, within a window of ± 2 weeks, were used. HTLV-I proviral load values were converted to copies per 100,000 PBMCs and log_10 _transformed prior to analysis. Data were analysed as average change from baseline to a given time point as measured by AUCMB using the T-test. All analyses were performed as randomised regardless of changes to study treatment during follow up. The study was funded by the departments of the participants. GlaxoSmithKline generously provided zidovudine and lamivudine for the study and matching placebos.

## Abbreviations

AUCMB Area under the curve minus baseline

CD Cluster of Differentiation

CTL Cytotoxic T- Lymphocyte

DNA Deoxyribonucleic acid

HAM HTLV-I-associated myelopathy

HTLV-I Human T-cell Lymphotropic Virus type I

IFN-γ Interferon-gamma

IL-2 Interleukin – 2

IQR Inter-Quartile Range

MCV Mean cell volume

MDR Multi-drug Resistance

PBMC Peripheral blood mononuclear cell

SD Standard Deviation

SE Standard Error

TNF-α Tumour necrosis factor – alpha

WHO World Health Organisation

## Competing interests

Graham P Taylor has received honoraria for teaching and travel grants from GlaxoSmithKline plc. No other conflicts of interest are declared.

## Authors' contributions

The study was conceived and designed by GPT, AB, JNW & CRMB; conducted by GPT, PG, YF, AB, AM, NN, PR, KU & MO, analysed by HG & AB and the manuscript was written by GPT, HG, AB, CRMB & JNW
